# Targeting mechanotransduction mechanisms and tissue weakening signals in the human amniotic membrane

**DOI:** 10.1038/s41598-019-42379-4

**Published:** 2019-04-30

**Authors:** David W. Barrett, Rebecca K. John, Christopher Thrasivoulou, Alvaro Mata, Jan A. Deprest, David L. Becker, Anna L. David, Tina T. Chowdhury

**Affiliations:** 1 Institute of Bioengineering, School of Engineering and Materials Science, Queen Mary University of London, Mile End Road, London, E1 4NS UK; 2 Department of Cell and Developmental Biology, University College London, Gower Street, London, WC1E 6BT UK; 3 Department of Obstetrics and Gynaecology, University Hospitals Leuven, Leuven, Belgium; 4 Lee Kong Chian School of Medicine, Nanyang Technological University, 11 Mandalay Road, Singapore, 308232 Singapore; 5 Institute for Women’s Health, University College London, 86-96 Chenies Mews, London, WC1E 6HX UK

**Keywords:** Paediatric research, Biomedical engineering

## Abstract

Mechanical and inflammatory signals in the fetal membrane play an important role in extracellular matrix (ECM) remodelling in order to dictate the timing of birth. We developed a mechanical model that mimics repetitive stretching of the amniotic membrane (AM) isolated from regions over the placenta (PAM) or cervix (CAM) and examined the effect of cyclic tensile strain (CTS) on mediators involved in mechanotransduction (Cx43, AKT), tissue remodelling (GAGs, elastin, collagen) and inflammation (PGE_2_, MMPs). In CAM and PAM specimens, the application of CTS increased GAG synthesis, PGE_2_ release and MMP activity, with concomitant reduction in collagen and elastin content. Co-stimulation with CTS and pharmacological agents that inhibit either Cx43 or AKT, differentially influenced collagen, GAG and elastin in a tissue-dependent manner. SHG confocal imaging of collagen fibres revealed a reduction in SHG intensity after CTS, with regions of disorganisation dependent on tissue location. CTS increased Cx43 and AKT protein and gene expression and the response could be reversed with either CTS, the Cx43 antisense or AKT inhibitor. We demonstrate that targeting Cx43 and AKT prevents strain-induced ECM damage and promotes tissue remodelling mechanisms in the AM. We speculate that a combination of inflammatory and mechanical factors could perturb typical mechanotransduction processes mediated by Cx43 signalling. Cx43 could therefore be a potential therapeutic target to prevent inflammation and preterm premature rupture of the fetal membranes.

## Introduction

Premature rupture of the fetal membranes (PROM) is a pathological process in which a tear develops in the amniotic membrane (AM) leading to tissue fragmentation and detachment of the chorioamnion from the uterine wall. Preterm PROM (PPROM) affects up to 40% of all preterm deliveries (less than 37 weeks gestation) and before the onset of labour^1^. PPROM is usually spontaneous and has a multifactorial aetiology related to inflammation, infectious processes or vaginal bleeding^2,3^. Uterine stretch and uterine contractions play an important role in PPROM^4^. Women are more likely to deliver preterm if they carry multiple pregnancies, have uterine structural anomalies that limit uterine size such a unicornuate uterus or there is excessive stretch of the FM due to polyhydramnios^5–8^. Previous studies have suggested an association between mechanical stretch and inflammatory factors, making it possible to counteract the intracellular process with pharmacological agents^9–11^. However, there is no consensus in this regard and the development of therapeutics to prevent FM weakening mechanisms are controversial.

A number of factors have been previously reported to disrupt the morphological, mechanical and extracellular matrix remodelling dynamics. The pathophysiological failure of the tissue is associated with increased inflammation involving upregulation of pro-inflammatory cytokines such as interleukins (IL-1β, IL-6, 8, 18 and TNFα) and proteolytic enzymes in the amniotic fluid and FM in patients with chorioamnionitis and PPROM^12–18^. In particular, TNFα and IL-1β have been reported to increase matrixmetalloprotease-9 (MMP-9) and PGE2 production in human chorioamnion and amnion epithelial cells leading to apoptosis^19,20^. Synergy between MMP activation and reduction in TIMP-1 was shown to reduce tensile strength due to disruption of the collagen fibril network, activation of apoptotic mechanisms and counter regulation of normal tissue remodelling mechanisms with the development of a FM weak zone^21–23^. Exposure of amniocytes to IL-1β or TNFα increased MMPs and PGE_2_ levels in a concentration-dependent manner and mimics the effects of oxidative stress mechanisms enhanced by reactive oxygen species (ROS) which increased collagen degradation and apoptosis^24–27^. Taken together, these biochemical changes influence the viscoelastic properties of the tissue. hindering its ability to withstand the impacts of repetitive mechanical stretch, eventually leading to membrane weakening and rupture. It is widely recognised that MMP-9 degrades collagen type IV, which is found at high levels in the basement membranes, suggesting that the AM plays a key role in overall membrane integrity^28–30^.

A number of *in vitro* model systems have been developed to examine the effect of repetitive stretch forces in regulating mechanotransduction processes in the FM^31,32^. For example, the application of 11% static stretch to human amnion epithelial cells activates NF-κB and COX-2 expression leading to enhanced production of PGE_2_, IL-1β, IL-6 and IL-8 after 6 hr^33,34^. IL-8 was reported to increase in human FM and decidua following mechanical stretch in a time and load-dependent manner^35^. Our group has previously shown that repetitive cyclic tensile strain (2% CTS) increased the expression of connexin 43 (Cx43), also known to be upregulated in the myometrium following uterine stretch^36–39^. The increase in Cx43 expression in the AM was associated with enhanced COX-2 gene expression, PGE_2_ release, and glycosaminoglycan (GAG) content, concomitant with a reduction in collagen and elastin content, suggesting an important role for mechanical and inflammatory factors in tissue weakening. In a nonhuman primate model, uterine overdistension was reported to increase production of TNFα, PGE_2_, IL-6, IL-8 and CCL2 in amniotic fluid leading to tissue remodelling in the AM and myometrium and preterm birth^5^. The small increases in FM stretch could initiate the FM weakening pathways due to non-recoverable deformation with continued cycles of high force stretching. We could speculate that the collagen fibres could realign after repetitive stretch as a protective mechanism to prevent rupture, but long-term repetitive stretching will lead to tissue failure^38^. Whilst, the previous models did not consider the dynamic nature of the collagen remodelling network and its fibre organisation in membranes overlying the cervix (CAM) or placenta (PAM), the present study examined the effect CTS on mediators involved in mechanotransduction (Cx43, AKT), tissue remodelling (GAG, elastin, collagen) and inflammation (PGE_2_, MMPs) in AM from CAM and PAM locations and explores a therapeutic approach to prevent tissue weakening and repair.

## Results

### Characterisation of collagen orientation in human AM subjected to CTS

**Figure 1:**
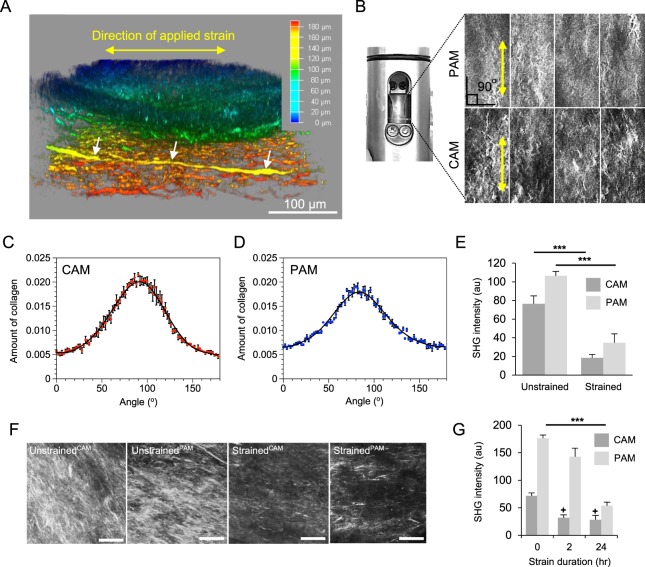
Characterization of collagen alignment and microscopic distribution in human amniotic membranes subjected to cyclic tensile strain. Collagen fibre alignment in specimens from the cervical amniotic membrane (CAM) or placental regions (PAM) was confirmed by second harmonic generation (SHG) imaging. Yellow arrow in (**A**) show direction of applied strain and collagen fibre alignment by SHG confocal microscopy (**B**) after application of cyclic tensile strain (2% CTS, 1 Hz) with the angle of orientation clearly distributed in one direction (**C**,**D**). Comparison of SHG intensity values in CAM and PAM specimens after application of CTS for 24 hr are shown in (**E**). Representative SHG confocal images are shown in (**F**) for n = 16 replicate fields of view from two donors (scale bar = 100 µm). Temporal changes in SHG intensity analysis after application of CTS for 0, 2 and 24 hours in CAM and PAM specimens are shown in (**G**). Error bars in (**E**,**G**) represent the mean and SEM values of n = 16 replicates from four donors.

To characterise the direction of collagen fibre alignment, CAM and PAM specimens were examined by SHG imaging and quantified by collagen orientation distribution analysis (Fig. 1). Representative SHG images of CAM and PAM specimens subjected to cyclic tensile strain (2% CTS, 1 Hz) for 24 hr showed evidence of collagen fibres that are much more organised and appeared dense, elongated and highly aligned (yellow arrows, Fig. 1A,B). More specifically, there was a region of highly polarised fibres aligned in the direction of applied strain at approximately ∼90° in strained CAM and PAM specimens (Fig. 1C,D, respectively). However, quantification of collagen fibres revealed a significant reduction in SHG intensity after CTS in CAM and PAM specimens when compared to unstrained control specimens (both p < 0.001; Fig. 1E). Analysis by SHG imaging showed that the organisation degree of collagen fibres varied and was dependent on the microscopic regions of the tissue with intense collagen disorganisation in strained CAM compared to strained PAM specimens than the highly aligned fibres in unstrained controls (Fig. 1F). In particular, values for SHG intensity in strained CAM specimens were significantly reduced after application of CTS for 2 and 24 hr when compared to time = 0 (both p < 0.05; Fig. 1G). In contrast in strained PAM specimens, CTS application was time-dependent with a greatest reduction in SHG intensity values at 24 hr (p < 0.001; Fig. 1G) than 2 hr when compared to time = 0 hr.

### The effect of CTS and Cx43/AKT inhibition on tissue remodelling factors

**Figure 2:**
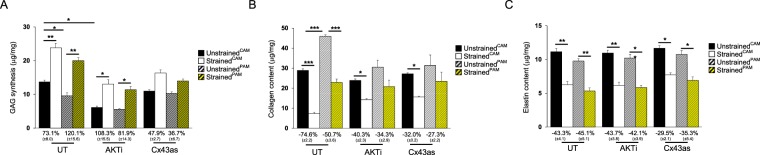
The effect of cyclic tensile strain and Cx43/AKT inhibition on extracellular matrix remodelling factors. Term human amniotic membranes isolated from the cervix (*C*AM) or placenta (*P*AM) regions were subjected to cyclic tensile strain (2% CTS, 1 Hz) for 24 hours, in the presence and absence of 0 or 25 µM AKTi or 50 µM Cx43 antisense (Cx43as). Absolute values for GAG synthesis (**A**), collagen (**B**), and elastin content (**C**) are presented in unstrained and strained AM specimens. The corresponding normalised strained values were expressed as a percentage change of the unstrained controls, with SEM values shown in brackets. Error bars represent the mean and SEM values of 16 replicates from four separate donors, where (*, ** or ***) indicates significant comparisons for unstrained and strained CAM or PAM conditions. All other comparisons (not indicated) were not significantly different.

Figure 2 examines the effect of CTS on GAG, elastin and collagen levels in CAM and PAM specimens. In unstrained controls, GAG synthesis was significantly higher in CAM (13.7 μg/mg) than PAM (9.5 μg/mg) specimens (p < 0.05; Fig. 2A). The application of CTS significantly increased GAG synthesis in CAM (73.1%) and PAM (120.1%) specimens when compared to unstrained controls (both p < 0.01; Fig. 2A). In unstrained specimens, stimulation with the AKT inhibitor (AKTi) alone significantly reduced GAG synthesis in CAM (p < 0.05) but not PAM specimens. Co-stimulation with CTS and AKTi significantly increased GAG synthesis (both p < 0.05) with a greater magnitude of stimulation in CAM (108.3%) than PAM specimens (81.9%). In contrast, the magnitude of stimulation was reduced in CAM (47.9%) and PAM specimens (36.7%) after co-stimulation with CTS and the Cx43 antisense (Cx43as).

In unstrained specimens, collagen content was significantly higher in PAM (45.9 μg/mg) compared to CAM (28.8 μg/mg) specimens (p < 0.001; Fig. 2B). The application of CTS significantly reduced collagen content in CAM and PAM specimens (both p < 0.001; Fig. 2B), with a greater magnitude of inhibition in CAM (−74.2%) than PAM (−50.6%) specimens. In unstrained specimens, stimulation with either the AKTi or Cx43as alone did not significantly affect collagen content in CAM or PAM specimens. However, co-stimulation with both CTS and either the AKTi or Cx43as significantly reduced collagen content in CAM specimens (both p < 0.05, Fig. 2B) but not PAM specimens.

In unstrained controls, the levels of elastin content were broadly similar for CAM and PAM specimens with values ranging from 9.8 μg/mg to 11.1 μg/mg (Fig. 2C). The application of CTS significantly reduced elastin content in CAM and PAM specimens when compared to unstrained controls (both p < 0.01; Fig. 2C), with the magnitude of inhibition broadly similar for CAM (−43.3%) and PAM specimens (−45.1%). In unstrained specimens, stimulation with either the AKTi or Cx43as alone did not significantly affect elastin content in CAM or PAM specimens. Co-stimulation with both CTS and either the AKTi or Cx43as significantly reduced elastin content in CAM or PAM specimens (either p < 0.05 or p < 0.01; Fig. 2C). The magnitude of inhibition was broadly similar for CAM (−43.7%) and PAM specimens (−42.1%) co-stimulated with CTS and AKTi. In contrast, the magnitude of inhibition was reduced for CAM (−29.5%) and PAM specimens (−35.3%) after co-stimulation with CTS and the Cx43as.

### The effect of CTS and Cx43/AKT inhibition on inflammatory factors

**Figure 3:**
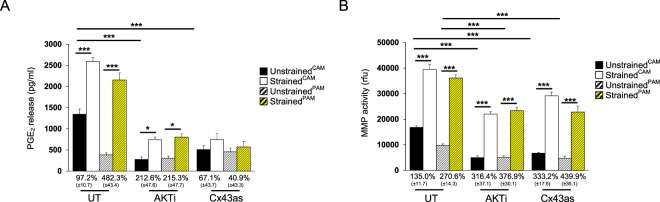
The effect of cyclic tensile strain and Cx43/AKT inhibition on inflammatory mediators. Term human amniotic membranes isolated from the cervix (*C*AM) or placenta (*P*AM) regions were subjected to cyclic tensile strain (2% CTS, 1 Hz) for 24 hours, in the presence and absence of 0 or 25 µM AKTi or 50 µM Cx43 antisense (Cx43as). Absolute values for PGE_2_ release (**A**) and MMP activity (**B**) are presented in unstrained and strained AM specimens. The corresponding normalised strained values were expressed as a percentage change of the unstrained controls, with SEM values shown in brackets. Error bars represent the mean and SEM values of 16 replicates from four separate donors, where (*, ** or ***) indicates significant comparisons for unstrained and strained CAM or PAM conditions. All other comparisons (not indicated) were not significantly different.

Figure 3 examines the effect of CTS in combination with either the Cx43as or AKTi on PGE_2_ release and MMP activity in CAM and PAM specimens. In unstrained controls, PGE_2_ release was significantly higher in CAM (1345.1 μg/ml) than PAM (382.9 μg/ml) specimens (p < 0.001; Fig. 3A). The application of CTS significantly increased PGE_2_ release in CAM and PAM (both p < 0.001, Fig. 3A), with the magnitude of stimulation greater in PAM (482.3%) than CAM (97.2%) specimens. In unstrained CAM and PAM specimens, stimulation with either the AKTi or Cx43as alone significantly reduced PGE_2_ release compared to untreated control CAM and PAM specimens (both p < 0.001; Fig. 3A). Co-stimulation with CTS and the AKTi significantly increased PGE_2_ release in CAM and PAM specimens (both p < 0.05; Fig. 3A) but not with the Cx43as. The magnitude of stimulation after CTS was greater in CAM and PAM specimens treated with AKTi (212.6% and 215.3%, respectively) than Cx43as 67.1% and 40.9%, respectively).

In unstrained controls, MMP activity was significantly higher in CAM (16, 748.1 rfu) than PAM (9751.2 rfu) specimens (p < 0.001; Fig. 3A). The application of CTS significantly increased MMP activity in CAM and PAM (both p < 0.001, Fig. 3B), with the magnitude of stimulation greater in PAM (270.6%) than CAM (135.0%) specimens. In unstrained CAM and PAM specimens, stimulation with either the AKTi or Cx43as alone significantly reduced MMP activity compared to untreated control CAM and PAM specimens (both p < 0.001; Fig. 3B).

Co-stimulation with CTS and either the AKTi or Cx43as significantly increased MMP activity in CAM and PAM specimens (all p < 0.001; Fig. 3A). The magnitude of stimulation after CTS was broadly similar for CAM and PAM specimens treated with AKTi (316.4% and 376.9%, respectively). In contrast, the magnitude of stimulation was greater for PAM (439.9%) than CAM specimens (332.2%) after treatment with the Cx43as.

### The effect of CTS and pharmacological inhibition on Cx43 and AKT-1 gene expression

**Figure 4:**
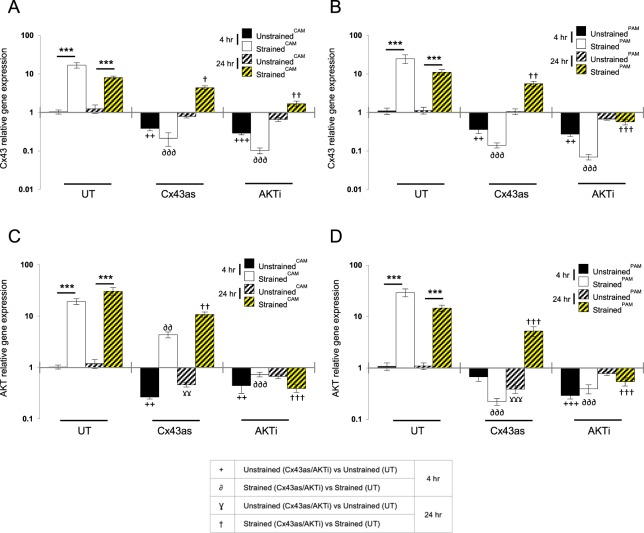
The effect of cyclic tensile strain and pharmacological agents on Cx43 and AKT-1 gene expression. Term human amniotic membranes isolated from the cervix (*C*AM) or placenta (*P*AM) regions were subjected to cyclic tensile strain (2% CTS, 1 Hz) for 4 hr and 24 hr, in the presence and absence of 0 or 25 µM AKTi or 50 µM Cx43 antisense (Cx43as). Gene expression of Cx43 and AKT in CAM (**A**,**C**) and PAM specimens (**B**,**D**) were presented as ratio values and normalised to unstrained control AM cultured in the absence of the chemical agent. In all cases, error bars represent the mean and SEM values of 10 replicates from three separate donors, where (*, ** or ***) indicates significant comparisons for unstrained and strained CAM or PAM conditions. All other comparisons (not indicated) were not significantly different.

Figure 4 examines the effect of CTS and either the Cx43as or AKTi on Cx43 and AKT-1 gene expression. In the absence of either the antisense or inhibitor, the application of CTS for 4 hr or 24 hr significantly increased Cx43 gene expression in CAM (both p < 0.001; Fig. 4A) and PAM (both p < 0.001; Fig. 4B) compared to unstrained controls. At 4 hr, stimulation with CTS alone or in combination with either the Cx43as or AKTi abolished Cx43 gene expression when compared to untreated CAM (p < 0.01 and p < 0.001; Fig. 4A) and PAM specimens (p < 0.01 and p < 0.001; Fig. 4B). At 24 hr, there was a marginal increase in Cx43 gene expression following co-stimulation with the Cx43as in CAM and PAM specimens but not with the AKTi.

In the absence of the antisense or inhibitor, the application of CTS for 4 hr or 24 hr significantly increased AKT-1 gene expression in CAM (both p < 0.001; Fig. 4C) and PAM (both p < 0.001; Fig. 4D) compared to unstrained controls. At 4 hr, stimulation with either the Cx43as or AKTi alone abolished AKT-1 gene expression when compared to untreated CAM (both p < 0.01; Fig. 4C) and PAM specimens (p < 0.01 and p < 0.001; Fig. 4D). However, co-stimulation with CTS and the Cx43as increased AKT-1 gene expression in CAM (p < 0.01; Fig. 4C) but not PAM specimens (Fig. 4D) and the response was increased at 24 hr. In contrast, stimulation with AKTi alone or in combination with CTS for 4 hr or 24 hr abolished AKT-1 gene expression.

### The effect of CTS and pharmacological inhibition on Cx43 and AKT protein levels

**Figure 5:**
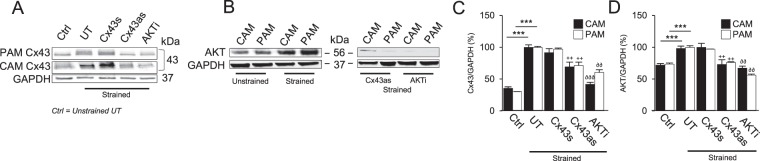
The effect of cyclic tensile strain and pharmacological agents on Cx43 and AKT protein expression in human amniotic membranes. Term human amniotic membranes from the cervical (CAM) or placental regions (PAM) were subjected to cyclic tensile strain (2% CTS, 1 Hz) for 24 hr, in the presence and absence of 0 or 25 µM AKTi, 50 µM Cx43 antisense (Cx43as) and/or 50 µM Cx43 sense oligodeoxynucleotides (Cx43s). Cx43 and AKT protein levels were examined in unstrained and strained CAM or PAM specimens by western blotting (**A**,**B**) and normalisation of Cx43 (**C**) or AKT (**D**) to GAPDH by densitometry analysis. Error bars represent the mean and SEM values for 4 to 9 replicates from three separate donors, where *p < 0.05; **p < 0.01 and ***p < 0.001.

Figure 5 examines the effect of CTS and either the Cx43as and AKTi on Cx43 (Fig. 5A) and AKT protein levels (Fig. 5B). In the absence of either the antisense or inhibitor, the levels of AKT and Cx43 protein levels in unstrained CAM and PAM specimens were broadly similar (Fig. 5C,D). The application of CTS significantly increased Cx43 (p < 0.001, Fig. 5C) and AKT (p < 0.001, Fig. 5D) protein levels in CAM and PAM specimens when compared to unstrained controls. Co-stimulation with CTS and either the Cx43as (Fig. 5A) or AKTi (Fig. 5B) reduced Cx43 and AKT protein expression in CAM and PAM specimens when compared to untreated controls (p < 0.01 and p < 0.001; Fig. 5C,D). In contrast, co-stimulation with CTS and the Cx43 sense (Cx43s) had no significant effect when compared to untreated control specimens.

## Discussion

**Figure 6:**
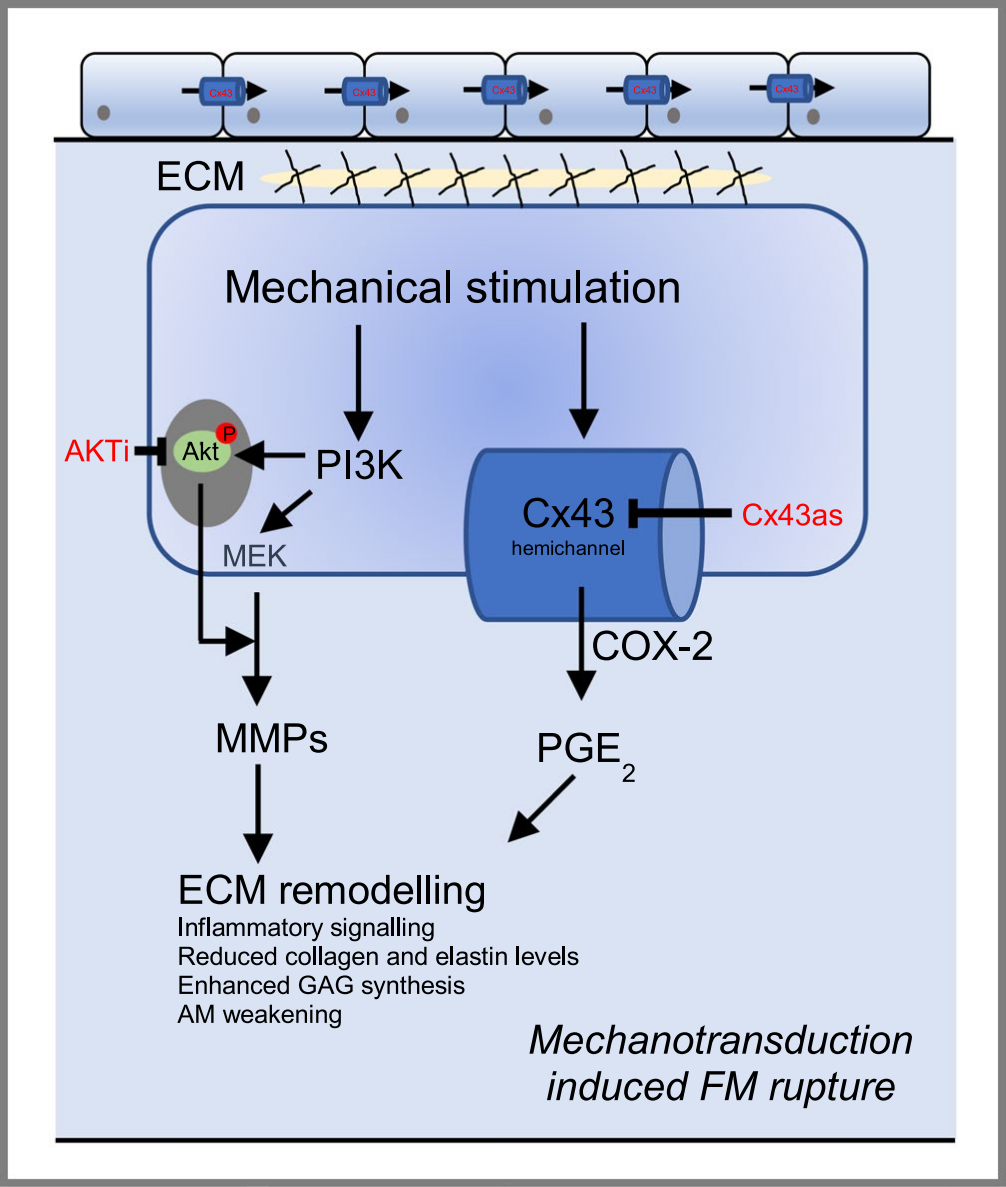
Key signaling events that lead to fetal membrane rupture following activation of stretch-activated pro-inflammatory pathways. The application of cyclic tensile strain (CTS) induced AKT and Cx43 protein expression leading to enhanced levels of PGE_2_ release and MMP activity. In contrast, CTS enhanced GAG synthesis which causes swelling and disruption of the extracellular matrix components via inhibition of collagen and elastin levels. An improved understanding of these mechanisms could lead to the further development of therapeutics for preventing PPROM.

The intracellular signalling mechanisms that promote FM weakening in response to mechanical stretch and inflammation remain unclear. Figure 6 summarises the potential interactions of mechanical stretch with the stretch-sensitive Cx43 and inflammatory targets. The CTS model was designed to enable investigation of pharmacological agents that could inhibit the intracellular mechanisms and examine the mechanisms leading to tissue weakening. With further study, the mechanical stretch model could be a useful tool to investigate therapeutics for AM repair and PPROM prevention.

### Potential mechanism of stretch-induced weakening in the AM

We investigated the effect of repetitive CTS on AM weakening and targeted Cx43, AKT/PI3K and COX-2/PGE_2_ signalling and ECM composition in unstrained and strained CAM and PAM specimens. We identified increased protein and gene expression of Cx43 and AKT after CTS compared to unstrained control AM. This was concomitant with increased MMP activity, PGE_2_ release and GAG synthesis, whilst collagen and elastin content decreased after CTS. Cx43 is a stretch-sensitive gap junctional protein, previously shown to be overexpressed in the myometrium in response to mechanical stimulation^36^. Cx43 is known to comprise a Ser-373 substrate binding site for AKT and in osteocytes, mechanical stimulation was reported to activate AKT and Cx43 phosphorylation, enhance their interactions with α_5_ integrin and Cx43 hemichannel opening^40,41^. Mechanical stimulation has also been linked with increased Cx43 translocation to the cell membrane^42^. The enhanced protein and gene expression of Cx43, PGE_2_ release and MMP activity has been previously reported to degrade components in the extracellular matrix. Indeed, Sutcliffe *et al*. 2015 showed elevated levels of Cx43 protein expression in chronic wounds for patients with venous leg, diabetic foot or pressure ulcers^43^. The wounded tissues are chronically inflamed with high levels of MMP activity and a significant reduction in collagen and elastin levels was revealed by 2-photon imaging^44^. The loss of the extracellular matrix in the inflamed tissue is consistent with our findings that CTS induced protein and gene expression of Cx43 and AKT and enhanced PGE_2_/MMPs in human CAM and PAM specimens. We propose that an increase in the stretch-sensitive Cx43/AKT signalling pathways plays a major role in the production of inflammatory mediators PGE_2_ and MMP activity, leading to collagen and elastin degradation, and enhanced GAG synthesis. We hypothesise that disruption to the extracellular matrix network in response to CTS-induced Cx43 and AKT signalling plays a predominant role in determining tissue homeostasis and extracellular matrix dynamics. However, it is difficult to speculate whether the Cx43/AKT mechanisms may potentially be augmented in preterm tissues, since the feto-maternal conditions such as pre-eclampsia will contribute to additional factors involving infection, stress, inflammation, oxytocin and influence the signalling process.

### Targeting the mechanotransduction process in the AM

Targeting the Cx43, AKT/PI3K and COX-2/PGE_2_ signalling with antisense led to dynamic changes in ECM composition and the release of inflammatory factors such as PGE_2_ and MMPs, as illustrated in Fig. 6. Whilst repetitive CTS strain was observed to induce matrix damage of the AM, this effect was partially prevented by treatment with agents that knockdown Cx43, AKT/PI3K and COX-2/PGE_2_ signalling pathways. Typically, exposure to the antisense or pharmacological agents reduced GAG synthesis after CTS, whilst the opposite effect was found for collagen and elastin content and was partially reversed after CTS. In addition, PGE_2_ release and MMP activity was reduced after CTS in the presence of the antisense and pharmacological agents. However, major differences between CAM and PAM were evident. The CAM was isolated from a region also known as the zone of altered morphology (ZAM). The ZAM in physiological membranes is characterised by swelling of the collagenous layers in the amnion and thinning of the cell-dense chorionic trophoblast later with a reduced thickness of the overall FM. This specific area is also associated with increased MMP activity and cellular apoptosis prior to the onset of labour. We also report a reduced SHG intensity in CAM regions compared to PAM, which reduced further with repetitive CTS and influenced collagen organisation. It is difficult to know whether the collagen orientation identified in the current model mimics the *in utero* environment and identifies the need to develop better quantitative full thickness FM models for understanding weakening. Interestingly, this effect was supported by our data which shows enhanced PGE_2_, MMP activity, and GAG synthesis in AM specimens taken from the cervical region. This was also confirmed by reduced collagen content in this region of the AM.

Whilst the present study attempted to quantify collagen organisation, mimic ECM disruption and the tissue weakening signals as experienced by women during pre-labor, repeated stretch of the AM may not mimic the *in utero* situation. The development of *in vitro* bioreactor systems or organ on a chip models are highly challenging due to the complexity of the equipment needed to exert multiple types of mechanical forces on FM tissues. To date, no research groups have developed predictive mechanical systems that can incorporate large replicate numbers with micro-fluidic technologies and pharmacological agents to study cellular processes, mechanotransduction and weakening mechanisms in the FM. The present study examined the mechanisms in term placental membranes taken from only 28 donors, with low or moderate replicate numbers to compare the different variables and analyse by protein and gene expression. We did not address the differences in mechanical forces *in vitro* with the *in utero* environment, or the potential difference in the mechanisms for preterm with term placental membranes. A multi-disciplinary approach combining engineering with biology will help researchers understand the intracellular mechanisms of healing and repair as well as providing a physiological model in which to examine AM repair strategies are therefore needed.

### Differences in the effect of CTS on matrix composition in AM overlying the cervix or placenta

Interestingly, total GAG content during cervical ripening has been reported to increase with advancing gestational age and with parturition, concomitant with a reduction in collagen, where fibres appear thinner and more dispersed^45^. GAGs are long unbranched polysaccharides that contain negatively charged repeating disaccharide units. This property attracts Na^+^ ions, which creates a hydrostatic pressure leading to swelling of the tissue. Enhanced GAG content in AM overlying the cervix may affect the organisation of collagen and lead to increased tissue softening. Furthermore, the alterations in the AM matrix components were found to be accompanied with an increase in PGE_2_ and total MMP activity from tissues secreted by the CAM and PAM regions. PGE_2_ is known to increase in the cervix during cervical ripening and induces remodelling of cervical connective tissue by increased production of proteoglycans^46,47^. We report similar mechanisms following CTS with increased PGE_2_ levels and enhanced GAG synthesis, and in turn a reduction of collagen content mediated by overproduction of MMP activity demonstrated in PAM and CAM specimens subjected to CTS. This repetitive mechanical stimulation could activate stretch-sensitive proteins that initiate a pro-inflammatory response leading to overstimulation of the matrix degradation pathways. Furthermore, membranes from PAM regions were reported to be stronger than CAM due to greater cross-linking of the collagen network and specific patterns of tissue remodelling and membrane homogeneity. The differences in the mechanical integrity with tissue location could improve tolerance to the applied mechanical force, thereby affecting cellular processes, mechanotransduction and apoptosis. A better understanding of the mechanisms overlying the cervix and placenta will help to develop clinical treatments for targeted repair and healing of FM tissues after iatrogenic or spontaneous PPROM.

Kumar *et al*., previously reported the involvement of inflammation/infection and decidual bleeding/abruption pathways to FM weakening^48–50^. This group described an overlap between the two FM weakening pathways and by modelling inflammation using TNFα and placental abruption using thrombin. Induction of Granulocyte-macrophage colony-stimulating factor (GM-CSF) on the choriodecidual side leads to FM weakening. Interestingly, the previous study demonstrates that blocking the action of GM-CSF reduced FM weakening via inflammation and abruption pathways. Targeting this pathway using progestogen has been linked with a reduction in preterm birth when administered during pregnancy. However, the optimal progestogen is still under investigation^50^.

### Summary

We provide evidence that a combination of inflammatory and mechanical factors disrupt mechanotransduction processes mediated by abnormal Cx43/AKT signalling in the AM. However, further optimisation of antisense and pharmacological agents is required in combination with cyclic tensile strain for longer time periods. Combining these findings with rupture strength testing in the future will be beneficial in characterising how the perturbation of stretch-sensitive and pro-inflammatory pathways translates to changes in the biophysical properties of the FM.

## Methods

All methods were performed in accordance with the relevant guidelines and regulations at University College London Hospital and the School of Engineering and Materials Science, Queen Mary University of London.

### Amniotic membrane tissue isolation

Term human placentas were collected after women gave informed consent from women undergoing elective caesarean section (n = 28 separate donors, 37 to 42 weeks of gestation) at University College London Hospital. Ethical approval was given by the Joint UCL/UCLH Committees and the Ethics of Human Research Central Office (05/Q0505/82). Women with placenta praevia, multiple pregnancy, antepartum haemorrhage, PPROM, fetal growth restriction, clinical chorioamnionitis, meconium, and maternal diabetes were excluded from the study. At Caesarean section after delivery of the baby but before delivery of the placenta, a sterile Babcock tissue clip was placed on the lower edge of the AM within the uterine incision to provide a landmark. The placenta was separated from the uterus by gentle cord traction and rinsed with Earle’s Balanced Salt Solution (EBSS) for 3 min to remove excess maternal blood (Sigma-Aldrich, Fancy Road, Poole, UK). The AM was separated from the chorionic membrane (CM) and placenta tissue using gentle traction. The orientation of the membrane to the placenta, incision line and cervix was noted throughout the procedure and the AM nearest the cervix was identified using a clip. AM specimens (30 × 30 mm) from the cervix (CAM) and placenta (PAM) regions were dissected from the tissue as described previously (Chowdhury B, 2014) and cultured with 1 ml Dulbecco’s modified Eagle’s medium (DMEM) supplemented with 5 µg/ml penicillin, 5 µg/ml streptomycin, 15 µg/ml ascorbate and 20% Fetal Calf Serum (FCS) prior to mechanical loading experiments.

### Effect of cyclic tensile strain in CAM and PAM specimens

A well characterised *ex-vivo* bioreactor system was used to apply CTS to *C*AM or *P*AM specimens, as previously described^38^. Briefly, dumbbell shaped specimens with widths in the gauge and shoulder regions of 10 mm and 25 mm respectively, were secured in an individual custom-made stainless steel loading chamber with a grip-to-grip distance of 10 mm. To characterise the direction of collagen alignment, the AM explants were excised in the direction of applied CTS as demonstrated by SHG imaging and collagen orientation distribution analysis (Fig. 1). Eight chambers were connected to a single actuator arm and secured within a BOSE loading frame (BOSE Corporation, Eden, Prairie, Minnesota, USA) housed in a humidified incubator at 37 °C (Fig. 1b). Each chamber was filled with 1 ml DMEM + 20% FCS in the presence and absence of the following pharmacological agents: 25 µM AKTi (selectively inhibits AKT-1/2 isoforms), 50 µM Cx43 antisense oligodeoxynucleotides (Cx43asODNs, inhibits Cx43 mRNA expression) and 50 µM sense oligodeoxynucleotides (Cx43sODNs, control). Strained AM specimens were subjected to CTS ranging from 0 to 2% in a sinusoidal waveform at a frequency of 1 Hz. CTS was employed in an intermittent regimen (1 min CTS followed by 9 min unstrained) over the 24 hr culture period, to simulate typical contraction cycles induced during the onset of labour^38^. Control *C*AM or *P*AM specimens remained unstrained and were cultured in the bioreactor system for 24 hours. At the end of the experiment, unstrained and strained amniotic membranes and the corresponding media samples were transferred separately into Eppendorf tubes and stored at −80 °C until biochemical and gene expression analysis could be performed. In addition, specimens were fixed in 4% paraformaldehyde (PFA) for 2 hr and stored in PBS at 4 °C prior to immunostaining, immunofluorescence confocal microscopy or analysis by second harmonic generation (SHG) imaging.

### RNA extraction, cDNA synthesis and real-time quantitative PCR

Total RNA was extracted from AM specimens using Trizol reagent and purified using RNeasy Mini Kit (Qiagen, Manchester, UK). RNA was treated with DNA-free DNase for 20 min (Ambion Applied Biosystems, Warrington, UK). A total of 200 ng RNA was reverse transcribed using Enhanced Avian RT First Strand cDNA synthesis kit with oligo(dT) 23 primer (Sigma Genosys, Cambridge, UK). For real-time RT-qPCR, each reaction was run in duplicate on a 96-well plate containing 5 μl SYBR green mastermix, 2.5 μl cDNA and 2.5 μl primer pairs. The following specific primer sequences were used: Cx43 sense: 5′-CTC GCC TAT GTC TCC TCC TG-3′, antisense: 5′-TTG CTC ACT TGC TTG CTT GT-3′; and AKT-1 sense: 5′-TCT ATG GCG CTG AGA TTG TG-3′, antisense 5′-CTT AAT GTG CCC GTC CTT GT-3′ (Sigma Genosys, Cambridge, UK). The StepOnePlus™ Real-Time PCR System (ThermoFisher Scientific) was used for real-time detection of PCR products. Thermocycling conditions were 95 °C for 2 min, followed by denaturation of 40 cycles at 95 °C for 15 s, annealing at 60 °C for 1 min, and extension at 72 °C for 1 min. PCR efficiencies for optimal primer pair concentrations were derived from standard curves (n = 3) by preparing a ten-fold serial dilution of cDNA from a sample that represented the control. The real-time PCR efficiencies (E) of amplification for Cx43/AKT-1 was defined according to the relation, E = 10[−1/slope]. The R^2^ value of the standard curve exceeded 0.99 and revealed efficiency values ranging from 1.98 to 2 (98 to 100%). Primer specificity was verified by examining the melting curve. Relative quantification of Cx43/AKT-1 was estimated by normalizing the target to the reference gene, GAPDH and to the calibrator sample by a comparative Ct approach. Ratios were expressed on a logarithmic scale (arbitrary units).

### Biochemical analysis

*C*AM and *P*AM specimens were digested in PBS supplemented with 10 mM L-cysteine and 10 mM EDTA, pH 6.5 for 60 min and incubated overnight at 37′C with 0.1 units/ml Papain for 1 hour at 60 °C. The subsequent biochemical analysis has been extensively described in detail^38^. GAG synthesis was determined using the 1, 9-dimethly-methylene blue dye-binding assay in digest and media AM specimens and the values normalised to DNA measured with the Hoechst 33258 method. For analysis of collagen content, AM specimens were digested with 1 mg/ml of pepsin in 0.5 M acetic acid at 4 °C overnight and determined by hydroxyproline assay. Elastin content was determined in AM specimens according to manufacturer’s instructions for the Fastin Elastin Assay (Biocolor Life Science Assays, Co Antrim, UK). Values for collagen and elastin content were normalised to dry weight obtained after lyophilisation of AM specimens. Total MMP activity was measured in media samples using a fluorogenic substrate assay. 25 µl sample was incubated with 2.5 µM amino-phenyl mercuric acetate (APMA) for 1 hour at room temperature to activate latent MMPs. Each sample was subsequently mixed with an equal volume of 10 µM Dnp-PChaGCHAK(Nma) fluorogenic MMP substrate, 50 µl buffer (500 mM HEPES, 100 mM CaCl_2_, 0.5% Brij-35, pH 7.0) in a 96-well plate (Enzo Life Sciences, Exeter, UK) and reactions measured at excitation and emission values of 340 and 440 nm, respectively. The change in fluorescence was calculated in the linear region of the kinetic assay for each sample between a period of 5 to 120 min at 37 °C. The levels of PGE_2_ release were determined in media samples by commercial ELISA assay (R&D Systems Europe Ltd, Abingdon, UK).

### Second Harmonic Generation and confocal imaging

Unstrained and strained CAM and PAM specimens were imaged in the AM tissue region by two photon imaging on a Leica TCS SP8 acousto-optic beamsplitter (AOBS) multiphoton confocal laser scanning microscope (Leica, Milton Keynes, UK) with a Coherent Chameleon Ultra, Ti Sapphire mode-locked IR laser (Coherent UK Ltd, Cambridge UK), as previously described^51^. Briefly, samples were imaged with a 25x, 0.95 NA water-immersion objective. Collagen SHG signal was collected via the transmission detector and 430–450 nm barrier filter with a pump wavelength of 880 nm at 80 fs pulse width. Approximately 150 μm volumes were acquired through the full thickness of the AM at 1.5 μm z-section intervals. Parameters for laser power, detector gain and offset were kept constant for each sample so that direct comparisons of the 8 bit digital images could be made per patient to permit quantification^43,51^.

### SHG quantification

To characterise the direction of collagen alignment, an orientation distribution analysis using the Directionality ImageJ plug-in (v2) was performed. The Directionality plug-in calculates the spatial frequencies within an image given a set of radial directions. The method generated normalised histograms revealing the amount of fibres present between 0° and 180° with a bin size of 1°. SHG images were converted to binary and 2D orientation analysis calculated using the local gradient orientation method^51^.

### Western blotting

Unstrained and strained CAM and PAM specimens were snap frozen in liquid nitrogen prior to homogenisation using the Mikro Dismembrator U (Sartorious) for 2 min at 2000 rpm. The resulting tissue powder was resuspended in 100 μL RIPA lysis buffer (Sigma). To ensure efficient homogenization, the lysate was then triturated through a 21 G needle with a 1 mL syringe up to 5 times. The lysate was then centrifuged for 15 minutes at 14,000 rpm and the supernatant containing the soluble protein fraction was collected and protein concentration determined with the BCA assay (Pierce 23227). 20 μg of protein was loaded onto Precast gels for SDS-PAGE (Bio-Rad), transferred onto nitrocellulose membrane, stained in 0.1% Ponceau S for 2 min and washed in PBS + 0.1% Tween for 5 min. The membranes were blocked with 5% BSA for 1 hour on a roller mixer at room temperature and incubated with primary antibodies for Cx43 (1:8000, Sigma C6219, rabbit), AKT-1/2/3 (1:1000, Abcam 126811, rabbit) or GAPDH (1:10, 000, Abcam 8245, mouse). Membranes were washed and incubated with secondary antibodies IRDye 800CW (1:10 000, Donkey anti-rabbit IgG, LI-COR 926–32213, Molecular Probes, Life Technologies) or IRDyge 680RD (1:10, 000, Goat anti-mouse IgG, LI-COR 926-68070) with 0.1% PBS + 0.1% Tween and 5% BSA on a roller mixer for 1 hour in the dark at room temperature. The membranes were washed twice in PBS and 0.1% Tween for 10 min and once in PBS for 10 min. Protein blots were scanned using the LI-COR Odyssey infrared imaging system via infrared fluorescence and the Odyssey software was used for quantitative analysis of protein bands. Integrated intensity values were exported into Excel and the data normalised to endogenous control values.

### Statistical analysis

For the bioreactor studies, biochemical data represent the mean and SEM values of 8 to 16 replicates from four to six separate experiments, as indicated in the figure legend. Statistical analysis was performed by a two-way analysis of variance (ANOVA) and the multiple *post hoc* Bonferroni corrected *t*-tests to compare differences between unstrained and strained CAM and PAM specimens with treatment groups as indicated in the figure legend. In all cases, a level of 5% was considered statistically significant (p < 0.05). For analysis by western blot, error bars represent the mean and SEM values for 9 replicates from three separate experiments, where *p < 0.05; **p < 0.01 and ***p < 0.001.

## Supplementary information

Supplementary information
