# Supplementary material for: Targeting mechanotransduction mechanisms and tissue weakening signals in the human amniotic membrane
Source: Sci Rep. 2019 Apr 30;9:6718. doi: 10.1038/s41598-019-42379-4 (PMC6491562; doi:10.1038/s41598-019-42379-4)
Supplement: Supplementary file 1 — Supplementary information [file 41598_2019_42379_MOESM1_ESM.pdf]

Targeting mechanotransduction mechanisms and tissue weakening signals in the human amniotic membrane

David W. Barrett[1], Rebecca K. John[1], Christopher Thrasivoulou[2], Alvaro Mata[1], Jan A. Deprest[3], David L. Becker[4], Anna L. David[5], Tina T. Chowdhury[1,*]

[1]Institute of Bioengineering, School of Engineering and Materials Science, Queen Mary University of London, Mile End Road, London E1 4NS, UK.
[2]Department of Cell and Developmental Biology, University College London, Gower Street, London WC1E 6BT, UK.
[3]Department of Obstetrics and Gynaecology, University Hospitals Leuven, Leuven, Belgium.
[4]Lee Kong Chian School of Medicine, Nanyang Technological University, 11 Mandalay Road, Singapore, 308232.
[5]Institute for Women's Health, University College London, 86-96 Chenies Mews, London WC1E 6HX, UK.

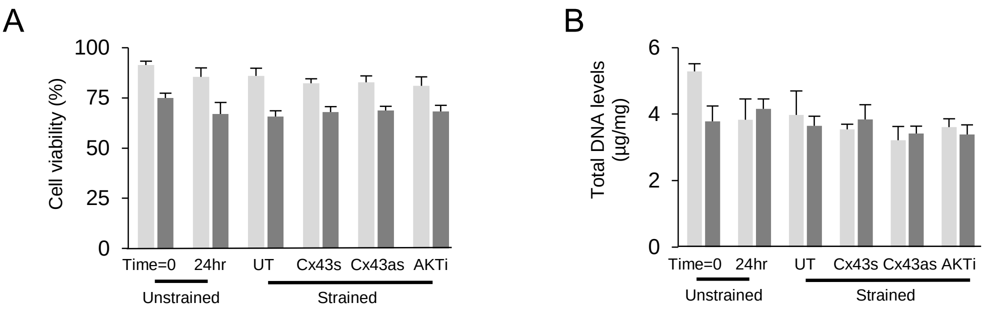

Supplementary Figure 1. The effect of cyclic tensile strain on cell viability (A) and total DNA levels (B) in human amniotic membranes cultured with pharmacological agents which inhibit Cx43 and AKT. Term human amniotic membranes were subjected to cyclic tensile strain (2% CTS, 1Hz) for 24 hours, in the presence and absence of 0 or 25 µM AKTi or 50 µM Cx43 sense (Cx43s) oligodeoxynycleotides or 50 µM Cx43 antisense (Cx43as) oligodeoxynycleotides. At the end of the experiment, CAM and PAM specimens were incubated with 5 mM of calcein AM and 5 mM ethidium homodimer for 45 min at 37°C and visualized with a 20x objective using an epifluorescence microscope. Cells were labelled as green (live) and red (dead) by calcein AM and ethidium homodimer, respectively. Percentage cell viability was calculated from at least ten fields of view. Cell viability (%) and DNA levels were compared to AM explants at time = 0. Values in unstrained and strained AM specimens represent the mean and SEM values of 8 replicates from four separate donors. All comparisons were not significantly (NS) different. Cell viability (A) and total DNA levels (B) remained stable after application of CTS for 24 hr in the presence and absence of the Cx43 antisense and AKTi when compared to unstrained control specimens.

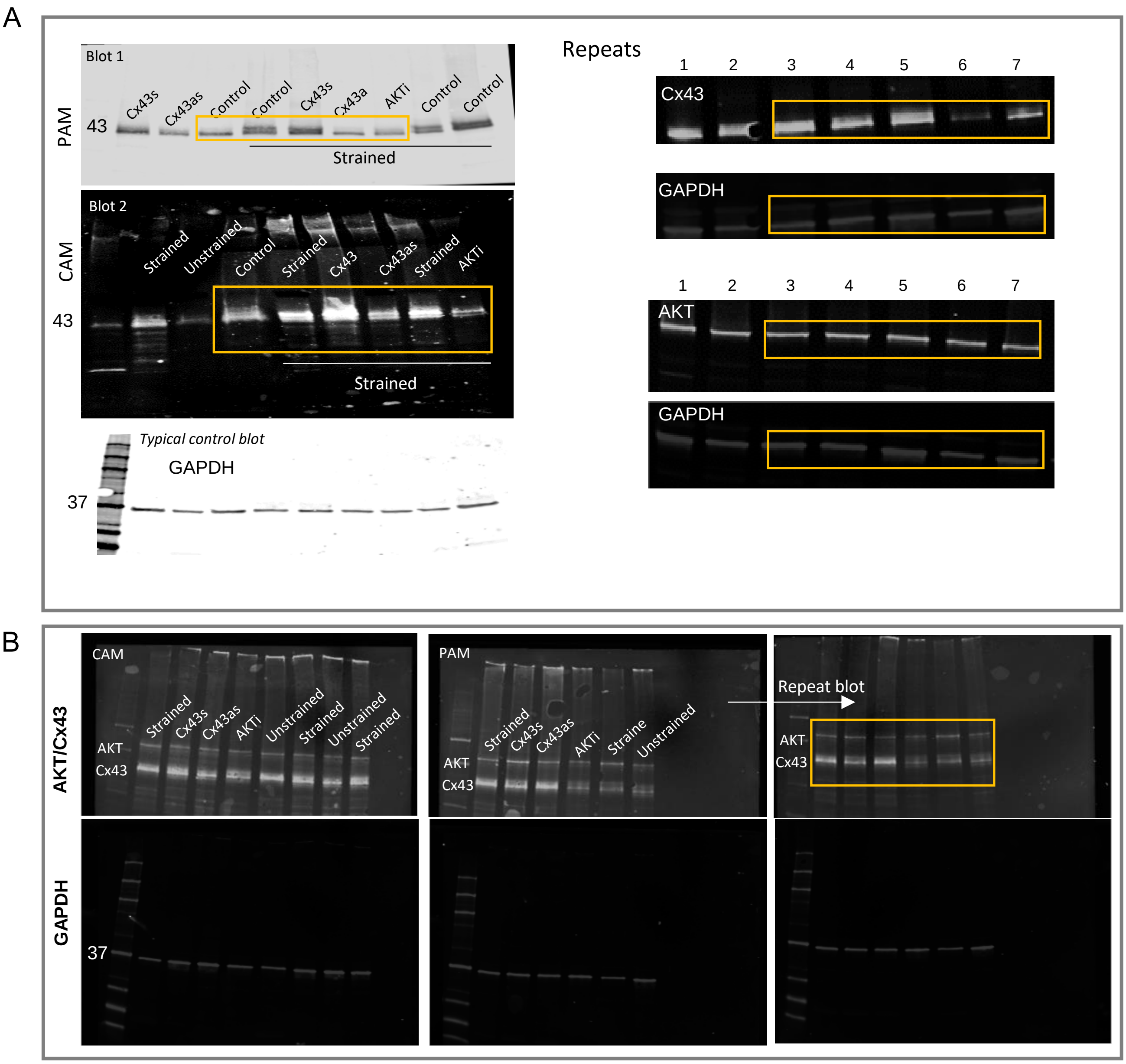

Supplementary Figure 2. The effect of cyclic tensile strain on Cx43 and AKT protein levels in human amniotic membranes. Term amniotic membranes (AM) from the cervical (CAM) or placental regions (PAM) were subjected to cyclic tensile strain (2% CTS, 1 Hz) for 24 hr, in the presence and absence of 0 or 25 μM AKTi, 50 μM Cx43 antisense (Cx43as) oligodeoxynycleotides or 50 μM Cx43 sense (Cx43s) oligodeoxynycleotides. Cx43 and AKT expression were examined in unstrained and strained CAM or PAM specimens by western blotting and normalisation to GAPDH. Representative protein blots with repeat blots for Cx43, GAPDH AKT are shown in (A) and (B). The original protein blot for Cx43, AKT and GAPDH without any changes to exposure for three repeats are also shown. Control membranes were cultured unstrained in the absence of the antisense or inhibitor.
